# PAQR3 suppresses the proliferation, migration and tumorigenicity of human prostate cancer cells

**DOI:** 10.18632/oncotarget.9807

**Published:** 2016-06-03

**Authors:** Wenqiang Huang, Weiwei Guo, Xue You, Yi Pan, Zhenyang Dong, Gaozhen Jia, Chenghua Yang, Yan Chen

**Affiliations:** ^1^ Key Laboratory of Nutrition and Metabolism, Institute for Nutritional Sciences, Shanghai Institutes for Biological Sciences, Chinese Academy of Sciences, University of Chinese Academy of Sciences, Shanghai, 200031, China; ^2^ School of Life Sciences and Technology, Shanghai Tech University, Shanghai, 200031, China; ^3^ Department of Urology, Shanghai Changhai Hospital, Second Military Medical University, Shanghai, 200433, China; ^4^ Joint Center for Translational Research of Chronic Diseases, Shanghai Changhai Hospital, Second Military Medical University, Shanghai 2000433, China

**Keywords:** human prostate cancer, PAQR3, cell proliferation, cell migration, tumor xenograft

## Abstract

As a newly discovered tumor suppressor, the potential function of PAQR3 in human prostate cancer has not been demonstrated. In this study, we report that PAQR3 is able to inhibit the growth and migration of human prostate cancer cells both *in vitro* and *in vivo*. Overexpression of PAQR3 inhibits the proliferation of PC3 and DU145 cells by both MTT and colony formation assays. Consistently, knockdown of PAQR3 enhances the proliferation of these cells. In wound-healing and transwell assays, overexpression of PAQR3 reduces the migration of PC3 and DU145 cells, while PAQR3 knockdown increases it. In a tumor xenograft model, overexpression of PAQR3 suppresses tumor growth of PC3 cells *in vivo*, while PAQR3 knockdown promotes the tumor growth. PAQR3 is also able to inhibit serum-induced phosphorylation of AKT and ERK in both PC3 and DU145 cells. In addition, PAQR3 suppresses the expression of epithelial-mesenchymal transition (EMT) markers in PC3 cells. Collectively, these data indicate that PAQR3 has a tumor suppressive activity in human prostate cancer cells and may stand out as a potential therapeutic target for prostate cancers.

## INTRODUCTION

Prostate cancer is the most common malignancy among men and causes the death of hundreds of thousands of men each year worldwide [1]. Most patients with prostate cancer are diagnosed with localized disease. Therapeutic approach includes radical prostatectomy, radiation therapy or combined methods including concurrent androgen deprivation therapy (ADT) with radiotherapy. The growth and proliferation of prostate cancer is mainly dependent on androgens, and ADT is an effective approach of controlling prostate cancer by eliminating the level of androgens in the patients. However, almost all patients eventually develop resistance to androgen deprivation, turning into castration-resistant prostate cancer (CRPC) [2]. The majorities of the patients relapse and develop into CRPC and eventually die of metastatic disease [2, 3]. In agreement with the clinical observation, it was lately discovered that enzalutamide, an oral androgen-receptor inhibitor, could significantly reduce the risk of radiographic progression and death and delay the initiation of chemotherapy in men with metastatic prostate cancer [4]. Considering the complex nature of prostate cancer, discovery of new molecules that regulate and control the development of prostate cancer is of paramount importance in the field.

The progestin and adipoQ receptor family, named as PAQR, is a highly conserved protein family that is composed of 11 members, PAQR1 to PAQR11 [5]. PAQR3 was recently discovered as a new member of tumor suppressor that is deregulated in different types of human cancer including colon cancer, gastric cancer, bladder cancer, liver cancer, osteosarcoma, breast cancer, and laryngeal squamous cell carcinoma [6–12]. PAQR3, also named as RKTG for Raf kinase trapping to Golgi, is a seven-transmembrane protein that is a type III membrane protein specifically localized in the Golgi apparatus in mammalian cells [13, 14]. PAQR3 has been discovered to be a negative regulator of Raf-1 by sequestrating Raf-1 to the Golgi apparatus, then blocking Ras-Raf-MEK-ERK signaling pathway [13]. PAQR3 has a negative role in the regulation of angiogenesis of endothelial cells [15]. PAQR3 functionally interacts with p53 in cancer formation and epithelial-mesenchymal transition (EMT) [16]. PAQR3 also inhibits AKT activation by two mechanisms, *i.e*., by inhibiting signaling of G protein βγ-subunit and by inhibiting PI3K via spatial regulation of p110a subunit [17, 18]. However, it is currently unknown whether PAQR3 has a functional role in prostate cancer. In this study, we investigated the potential functions of PAQR3 in prostate cancer and revealed that PAQR3 inhibits cell growth, migration and tumor development of human prostate cancer cells.

## RESULTS

### PAQR3 suppresses cell proliferation of prostate cancer cells

**Figure 1:**
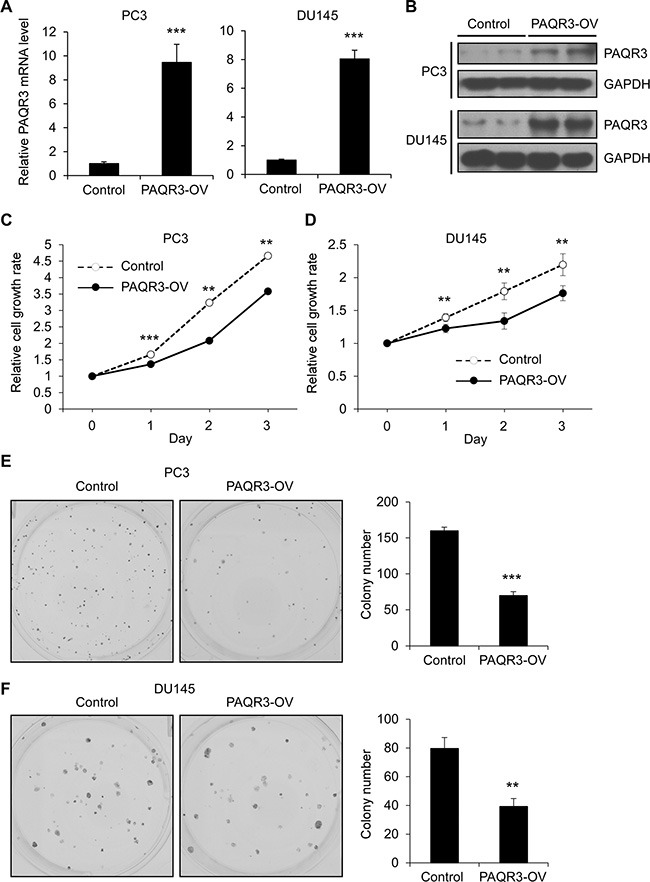
PAQR3 overexpression reduces cell proliferation and colony formation in human prostate cancer cells (**A**, **B**) The expression level of PAQR3 in control and PAQR3-overexpressing (PAQR3-OV) prostate cancer cells as detected by quantitative RT-PCR and Western blotting. (**C**, **D**) Effect of PAQR3 overexpression on the cell proliferation rate. PC3 or DU145 cells expressing empty vector or PAQR3 were cultured for 3 days and the cell proliferation rate was determined by MTT assay at the indicated time point. (**E**, **F**) Effect of PAQR3 overexpression on colony formation. PC3 or DU145 cells with stable expression of vector or PAQR3 were seeded into 6-well with 500 cells per well and cultured for 7 days, followed by crystal violet staining and colony counting. All the data are shown as mean ± SD and * for *P* < 0.05, ** for *P* < 0.01, *** for *P* < 0.001.

We investigated whether PAQR3 has a direct effect on the growth of human prostate cancer cells including PC3 and DU145 cell lines. We established stable cell clones with stable expression of PAQR3 via a lentivirus-based method. The expression of exogenous PAQR3 was confirmed by quantitative RT-PCR and Western blotting (Figure 1A and 1B). We next investigated the cell proliferation rate of these cells by MTT and colony formation assays. As shown in Figure 1C and 1D, the proliferation rate of PC3 and DU145 cells was significantly reduced by PAQR3 overexpression in comparison with the control cells. Consistently, colony formation was also significantly retarded by PAQR3 overexpression in PC3 and DU145 cells (Figure 1E and 1F).

**Figure 2:**
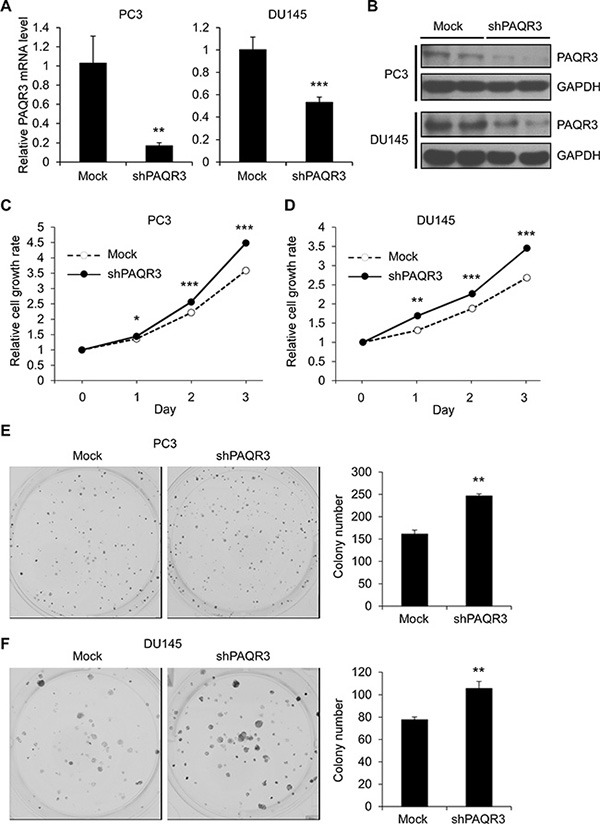
Knockdown of PAQR3 in human prostate cancer cells enhances cell proliferation and colony formation (**A**, **B**) The expression level of PAQR3 in PC3 and DU145 cells expressing control shRNA (mock) and PAQR3-specific shRNA (shPAQR3) were detected by quantitative RT-PCR and Western blotting. (**C**, **D**) Effect of PAQR3 knockdown on cell proliferation. The prostate cancer cells as in A and B were used to determine the cell proliferation rate by MTT assay at the indicated time point. (**E**, **F**) Effect of PAQR3 knockdown on colony formation. PC3 or DU145 cells as in A and B were seeded into 6-well with 500 cells per well and cultured for 7 days, and then used in crystal violet staining and colony counting. All data are shown as mean ± SD and * for *P* < 0.05, ** for *P* < 0.01, *** for *P* < 0.001.

We next analyzed the effect of PAQR3 knockdown on cell proliferation of the prostate cancer cells. Endogenous PAQR3 was silenced by a PAQR3-specific shRNA as previously reported [15]. The efficiency of PAQR3 knockdown was confirmed by RT-PCR and Western blotting (Figure 2A and 2B). We found that knockdown of PAQR3 was able to significantly enhance cell proliferation rate as well as colony formation (Figure 2C to 2F). Collectively, these data indicated that PAQR3 has a negative effect on the growth of human prostate cancer cells.

### Inhibition cell migration of prostate cancer cells by PAQR3

**Figure 3:**
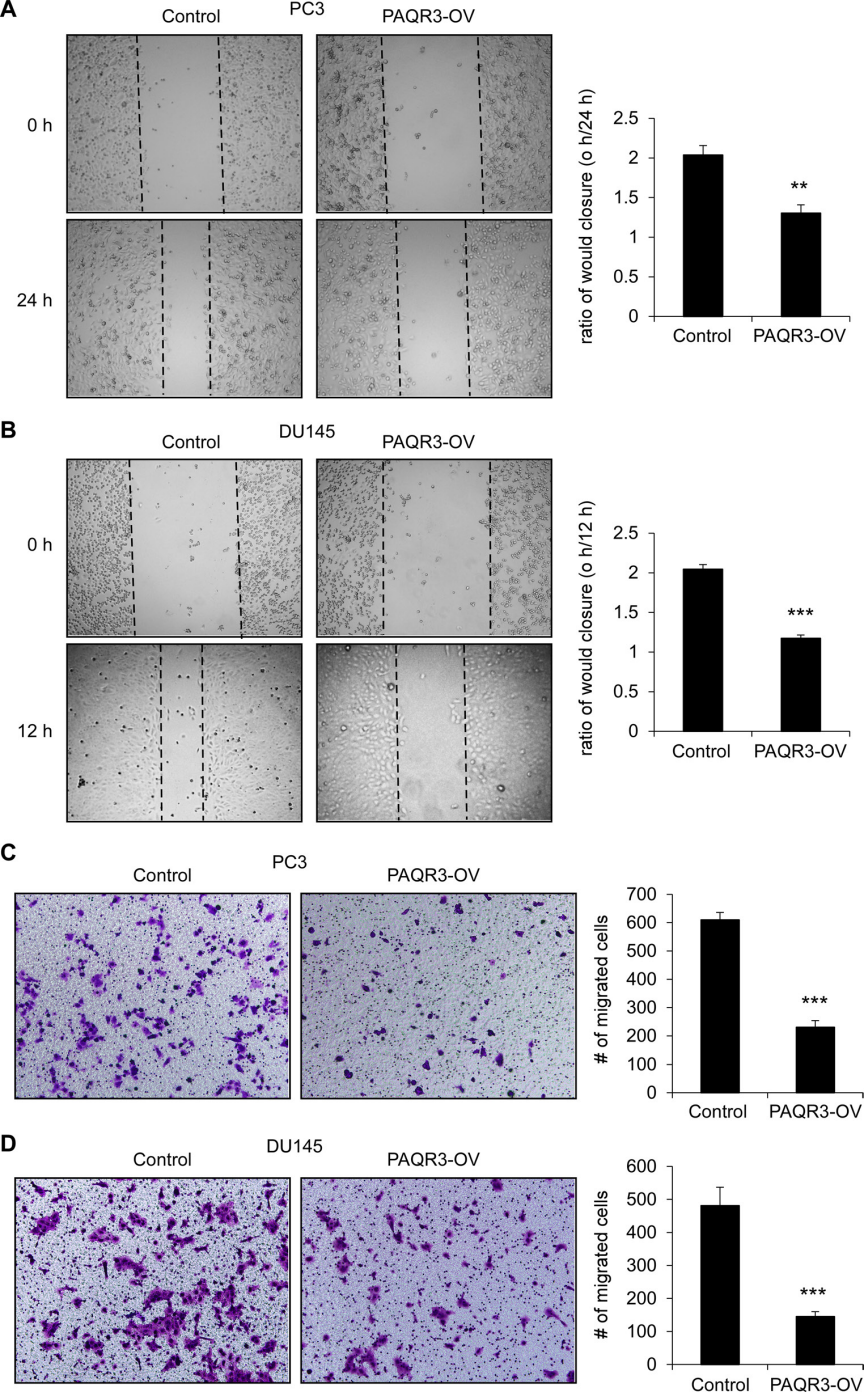
PAQR3 overexpression reduces migration of human prostate cancer cells (**A**, **B**) Effect of PAQR3 overexpression on cell migration in wound healing assay. A scratched-wound healing assay was performed with PC3 and DU145 cells stably expressing control vector or PAQR3 (PAQR3-OV). Photography was taken at 0 and 12 h (for DU145) or 24 h (for PC3) after the scratch and shown in the left panel. The ratio of wound closure were calculated and shown in the right panel. (**C**, **D**) Effect of PAQR3 overexpression on cell migration in transwell assay. PC3 or DU145 cells were seeded into transwell chambers that was set into 24-well plates. Then the cells on the lower chambers were fixed in 24 h (for DU145) or 48 h (for PC3) and stained with crystal violet, imaged and counted. Representative images of the staining are shown in the left panel and the statistic results are shown in the right panel. All the data are shown as mean ± SD and *** for *P* < 0.001.

**Figure 4:**
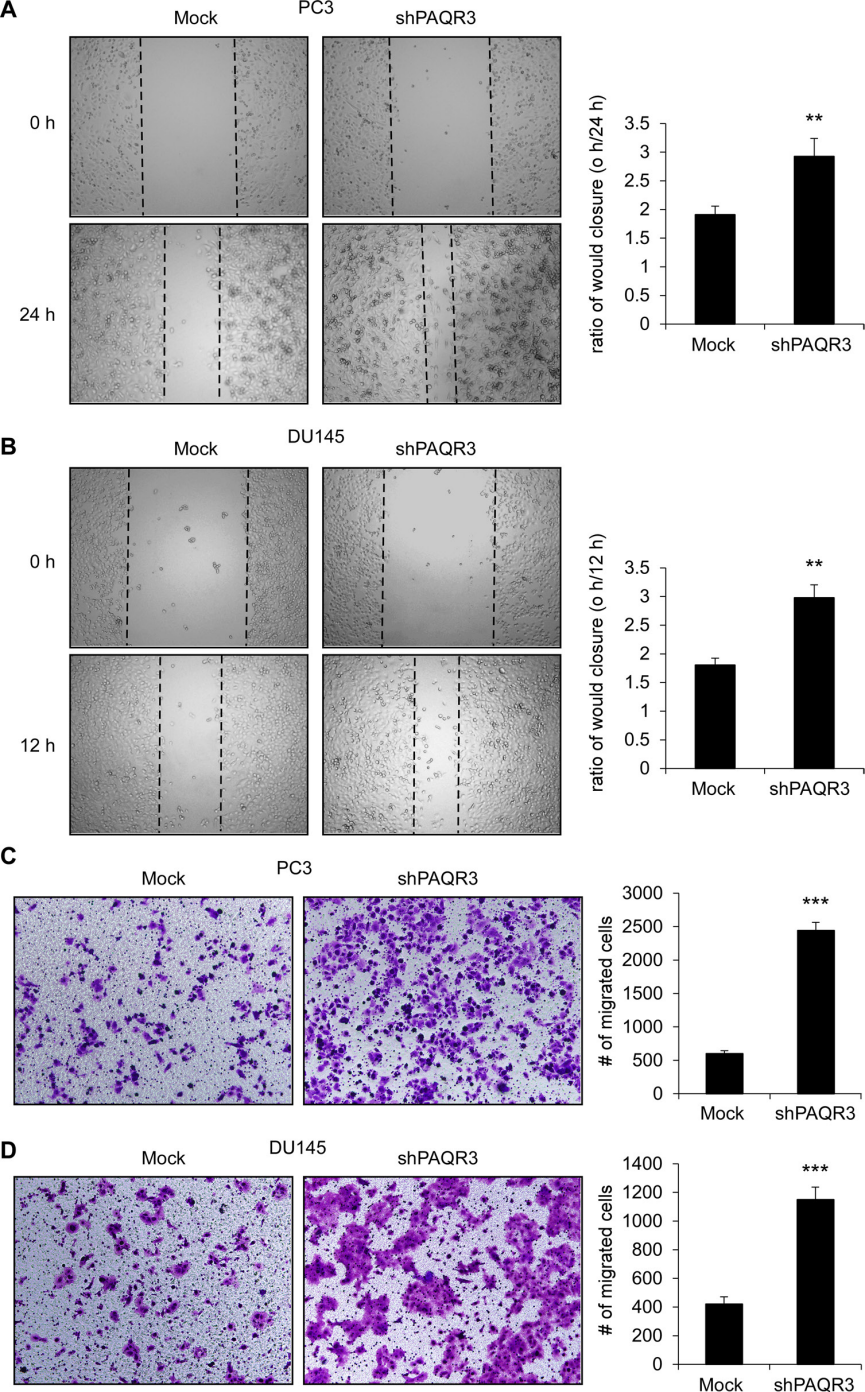
Knockdown of PAQR3 enhances migration in human prostate cancer cells (**A**, **B**) Effect of PAQR3 knockdown on cell migration in wound healing assay. A scratched-wound healing assay was performed with the cells expressing control or PAQR3-shRNA, followed by photography at the time as indicated.(**C**, **D**) Effect of PAQR3 knockdown on migration in transwell assay. PC3 or DU145 cells with expression of control shRNA (mock) or PAQR3-shRNA were seeded into transwell chambers. Then cells on small chambers were fixed in 24 h (for DU145) or 48 h (for PC3) and stained with crystal violet, imaged and counted. All the data are shown as mean ± SD and *** for *P* < 0.001.

We next investigated the possible function of PAQR3 on the migratory ability of prostate cancer cells. In a wound healing assay, overexpression of PAQR3 significantly reduced the migration rate of both PC3 and DU145 cells (Figure 3A and 3B). Consistently, the transwell assay further confirmed that the cell migration rate of these two prostate cancer cells was decreased by PAQR3 overexpression (Figure 3C and 3D). In contrast, the migration of the prostate cancer cells as analyzed by wound healing and transwell assays was significantly promoted by PAQR3 knockdown (Figure 4). These data, therefore, suggested that PAQR3 suppresses migration of prostate cancer cells.

### PAQR3 inhibits PI3K/AKT and MAPK/ERK signaling pathway and EMT features in prostate cancer cells

**Figure 5:**
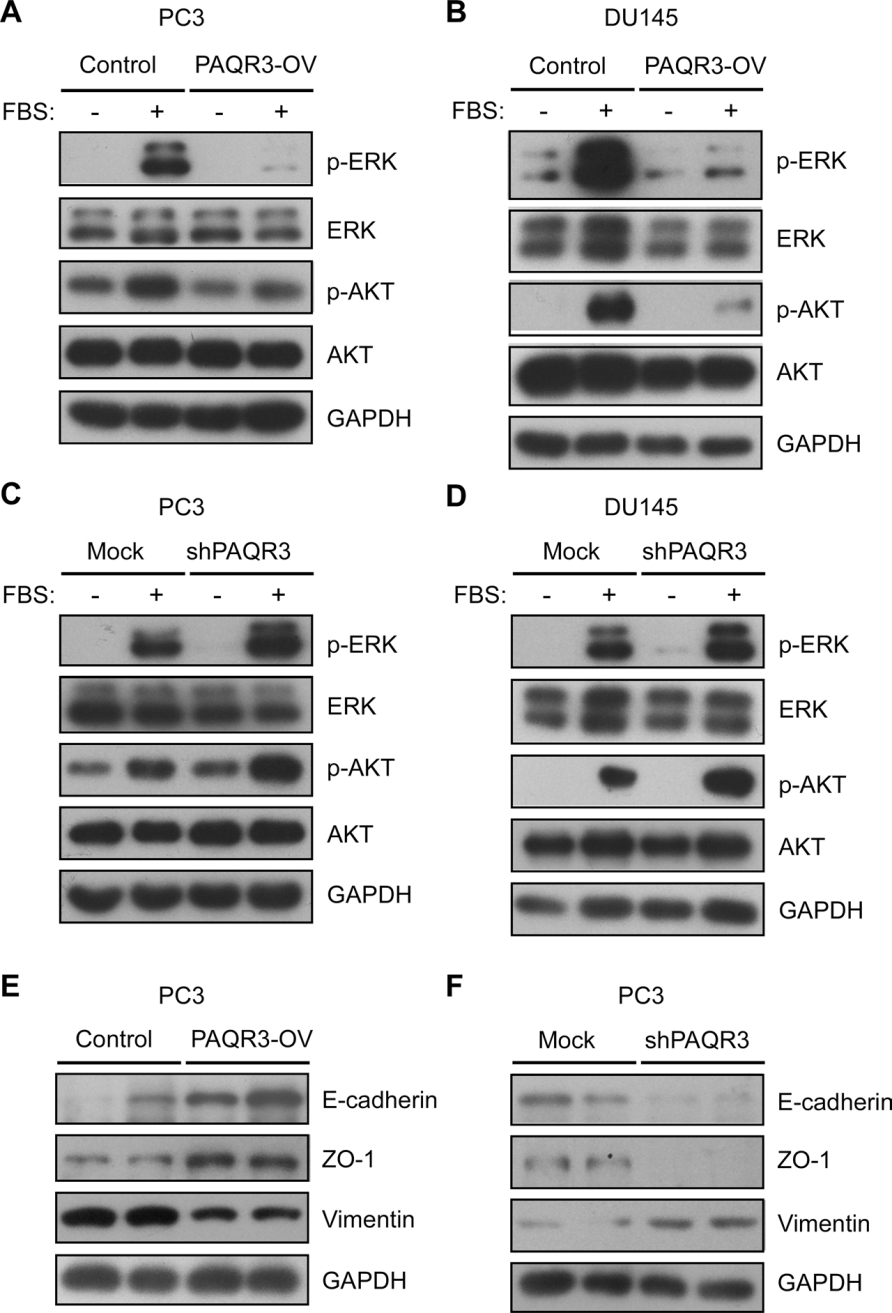
PAQR3 inhibits PI3K/AKT and MAPK/ERK signaling pathways and suppresses EMT features in human prostate cancer cells (**A**, **B**) PAQR3 overexpression inhibits PI3K/AKT and MAPK/ERK signaling pathways. After serum starvation for overnight, PC3 and DU145 cells with overexpression of control vector or PAQR3 (PAQR3-OV) were stimulated with 10% FBS for 15 min and the cells were harvested for immunoblotting using the antibodies as indicated. (**C**, **D**) Knockdown of PAQR3 enhances PI3K/AKT and MAPK/ERK signaling pathways. PC3 and DU145 cells that expressed control shRNA or PAQR3-specific shRNA (shPAQR3) were serum-starved overnight, followed by stimulation with 10% FBS for 15 min and immunoblotting. (**E**, **F**) PAQR3 suppresses EMT features. PC3 cells with overexpression or knockdown of PAQR3 were used to analyze the expression of epithelial markers E-cadherin and ZO-1 and mesenchymal marker vimentin.

Previous studies have revealed that PAQR3 is able to inhibit Ras/Raf/MEK/ERK and PI3K/AKT signaling cascades [13, 17, 18]. We next explored whether these two signaling pathways were also affected by PAQR3 in prostate cancer cells. As expected, addition of serum was able to stimulate Ras/Raf/MEK/ERK and PI3K/AKT signaling pathways shown as increased phosphorylation of ERK and AKT in both PC3 and DU145 cells (Figure 5 and Supplementary Figure S1). We found that the phosphorylation of ERK and AKT was robustly inhibited by PAQR3 overexpression in the prostate cancer cells (Figure 5A, 5B and Supplementary Figure S1). In contrast, knockdown of PAQR3 could enhance serum-induced phosphorylation of ERK and AKT (Figure 5C, 5D and Supplementary Figure S1). These data, therefore, indicated that PAQR3 is able to inhibit Ras/Raf/MEK/ERK and PI3K/AKT signaling cascades, likely explaining its negative effects on cell proliferation and cell migration (Figure 1 to 4).

We also analyzed the effect of PAQR3 on epithelial-mesenchymal transition (EMT), a critical step for tumor migration and metastasis [19]. In PC3 cells, overexpression of PAQR3 suppressed EMT features shown as increase of epithelial markers E-cadherin and ZO-1 with decrease of mesenchymal marker vimentin (Figure 5E). Consistently, knockdown of PAQR3 inhibited the expression of E-cadherin and ZO-1 while enhanced the expression of vimentin (Figure 5F).

### PAQR3 suppresses the growth of the prostate cancer *in vivo*

**Figure 6:**
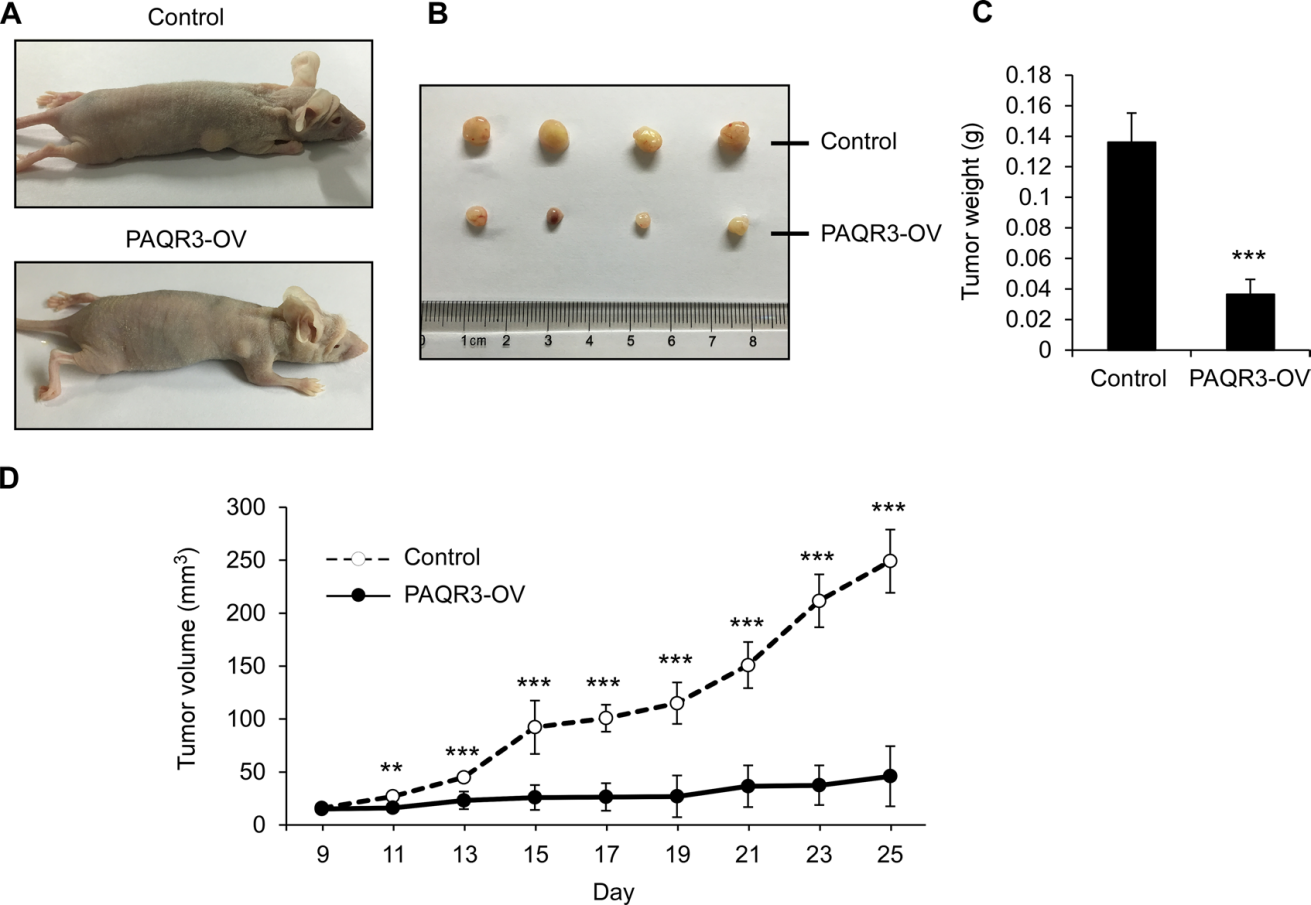
PAQR3 overexpression reduces the growth of PC3 cell xenografts in nude mice (**A**) Representative images of subcutaneous tumor xenografts in nude mice inoculated with PC3 cells expressing control vector or PAQR3. (**B**) Representative images of the tumors isolated from the mice. (**C**) The weight of the tumors. (**D**) Tumor volume as calculated according to the formula 0.5 × length × width^2^. All the data are shown as mean ± SD and ** for *P* < 0.01 and *** for *P* < 0.001.

**Figure 7:**
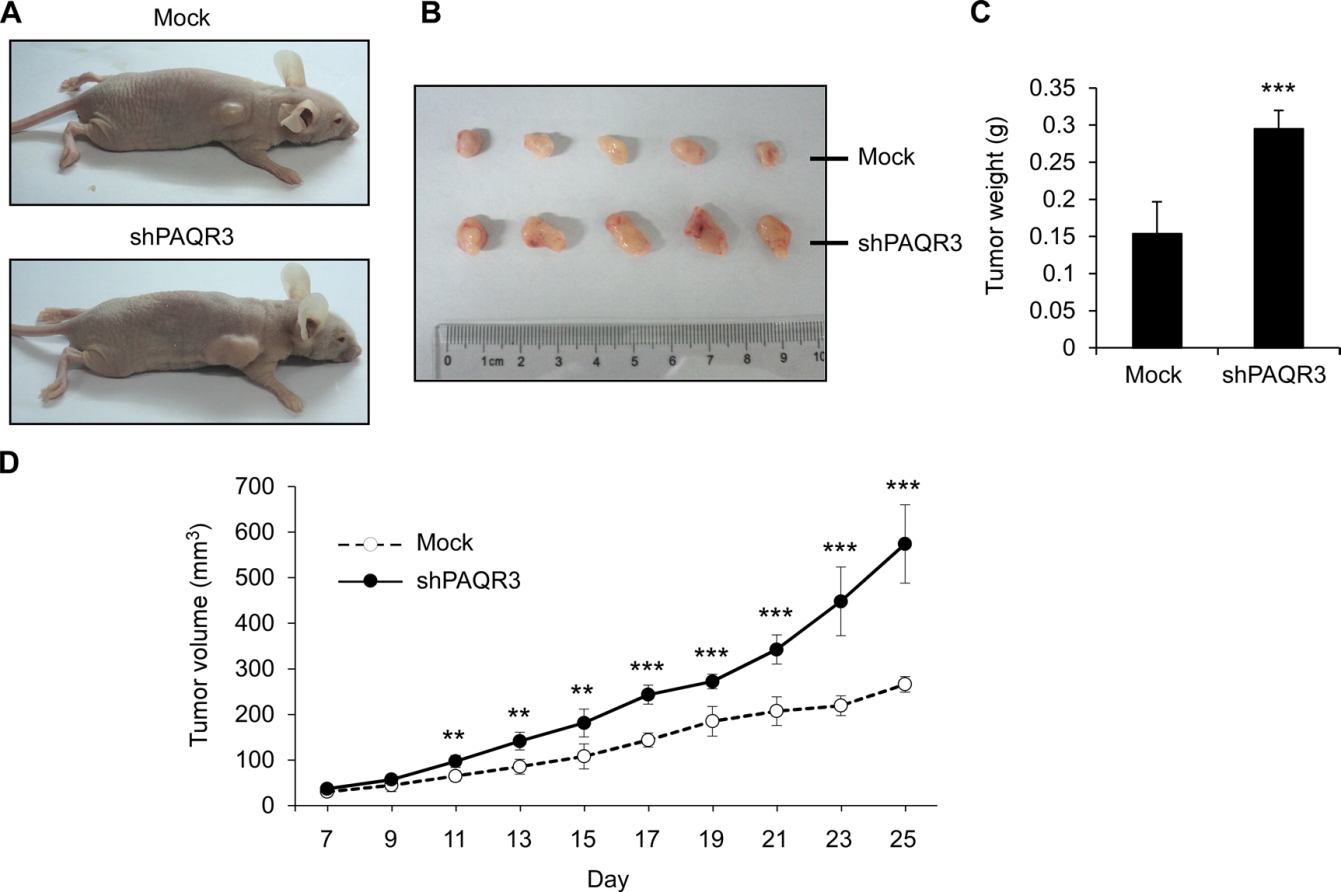
Knockdown of PAQR3 enhances the growth of PC3 cell xenografts in nude mice model (**A**) Representative images of subcutaneous tumor xenografts in nude mice inoculated with PC3 cells expressing control shRNA or PAQR3-specific shRNA. (**B**) Representative images of the tumors isolated from the mice. (**C**) The weight of the tumors. (**D**) Tumor volume as calculated according to the formula 0.5 × length × width^2^. All the data are shown as mean ± SD and ** for *P* < 0.01 and *** for *P* < 0.001.

In order to further elucidate the tumor suppressive activity of PAQR3 in prostate cancers, we next investigated the effects of PAQR3 on tumor growth using a xenograft model. PC3 cells with stable expression of either control vector or PAQR3 overexpression were implanted into the nude mice. The mice were sacrificed in 25 days when the tumor formation became obvious (Figure 6A). The growth of the prostate cancer cells in the mice as measured by tumor size, tumor weight and tumor volume was significantly reduced by PAQR3 overexpression (Figure 6B and 6C). We also investigated the effect of PAQR3 knockdown on tumor growth *in vivo*. The tumor size, tumor weight and tumor volume were all increased by PAQR3 knockdown in the mice (Figure 7). These observations, therefore, clearly indicated that PAQR3 has a powerful activity to suppress tumorigenicity of prostate cancer *in vivo*.

## DISCUSSION

Our studies have provided compelling evidence that PAQR3 functions as a tumor suppressor that controls the proliferation, migration and tumorigenicity of prostate cancer cells both *in vitro* and *in vivo*. At the cellular level, PAQR3 inhibits the cell proliferation, colony formation and migration of human prostate cancer cells. At the animal level, PAQR3 strongly suppresses the growth of prostate cancers in mice. Mechanistically, PAQR3 is able to inhibit both Ras/Raf/MEK/ERK and PI3K/AKT signaling cascades in prostate cancer cells and such inhibition likely underlies its tumor suppressive activity in these cells.

Due to potential significance of PAQR3 in regulating the tumorigenicity of prostate cancers, it will be important to further explore the functions of PAQR3 in the context of prostate cancer development. Firstly, it needs to be determined whether the expression level of PAQR3 is altered in human prostate cancer patient samples and if there is a change, whether the change is correlated with clinical parameters of the patients. To partially address this issue, we analyzed the publically available TCGA database. To date, there are 498 prostate adenocarcinoma samples and 52 normal prostate tissue samples in TCGA database. We found that the expression level of PAQR3 is significantly lower in prostate cancers than the normal tissues (Supplementary Figure S2), indicating that PAQR3 may function as a tumor suppressor in the development of human prostate cancers. Secondly, it will be important to further elucidate the molecular mechanisms associated with the tumor suppressive activity of PAQR3 in prostate cancers. Previous studies have revealed that PAQR3 is able to inhibit Ras/Raf/MEK/ERK and PI3K/AKT signaling cascades [13, 17, 18]. The suppressive activity of PAQR3 on these two pathways may partially underlie the inhibitory effect of PAQR3 on cell transformation and the initiation of tumorigenesis. In prostate cancers, androgen-mediated signaling pathway is crucial for the tumorigenicity of the tumor cells. It will be an interesting question to address whether PAQR3 is also involved in the regulation of androgen-mediated pathway. Thirdly, as PAQR3 is able to suppress migration of prostate cancers, the association of PAQR3 with metastasis and EMT need to be addressed in the future. It was discovered that the functional interaction of PAQR3 with p53 is implicated in EMT [16]. Lately, we found that PAQR3 is able to regulate ubiquitination and degradation of Twist1, a master regulator of EMT in gastric cancer cells [20]. It will be important to determine whether some key regulators of EMT are involved in PAQR3 regulation on migration and metastasis of prostate cancer cells. Finally, it will be of paramount significance to explore whether perturbation of PAQR3 function can be used as a new strategy to treat prostate cancers. In theory, elevation of the tumor suppressive activity of PAQR3 can stand out as an effective method to curb cancer formation. Discovery of chemicals or other means to mimic the tumor suppressive activity of PAQR3 will have double effects to control tumor cells, *i.e*., to inhibit tumor cell proliferation and to suppress tumor migration/metastasis. Future studies at these directions will undoubtedly bring new hopes to prostate cancer patients.

## MATERIALS AND METHODS

### Cell culture

HEK293T cell and Human prostate cancer cell lines PC-3, DU145 were obtained from the Institute of Cytobiology, Chinese Academy of Sciences. PC-3 cells were maintained in F12K medium (GIBCO) with 10% FBS (GIBCO). DU145 cell was cultured in MEM medium (GIBCO) with 10% FBS. HEK293T cell was maintained in DMEM medium (GIBCO) with 10% FBS. All cells were incubated in a cell incubator at 37^°^C under 5% CO2.

### Lentivirus packaging and infection

For lentiviral packaging, HEK293T cells (7 × 10^6^) were seeded in 15 cm cell culture dish, incubated for 24 h, and then transfected with 15 μg of lentivirus plasmids. The virus-containing medium was gathered and filtered through 0.45 μm filter (Millipore). Afterwards, the filtered supernatants were centrifuged at 20,000 g, 4°C for 2 h. The precipitate was suspended in 100 μl DMEM medium and sub-packaged in 1.5 ml tubes. For lentivirus infections, target cells were cultured into 6-well plates for infections by moderate virus-contained DMEM medium with polybrene (Sigma-Aldrich, 4 μg/ml), incubated at 37°C for 8 h and replaced by DMEM medium with FBS. After incubation for 36˜48 h, the infected cell lines were evaluated the infection efficiency of lentiviral by RT-PCR.

### RNA isolation and quantitative RT-PCR

The cells were lysed in Trizol reagent (Invitrogen). Total RNA was purified and reverse transcribed to cDNA according to the manufacturer's instructions. The quantification of target gene transcripts was detected by RT-PCR using SYBR Green Realtime PCR Master Mix (Toyobo) and ABI Prism 7900 sequence detection system (Applied Biosystems). The primers were as follows: Human PAQR3: 5′-CTCAAGGACAACCCGTACATCAC-3′and 5′-AAA CTTTTGATACACAGCCTGGAC-3′;

Huma β-actin: 5′-GATCATTGCTCCTCCTGAGC-3′ and 5′-ACTCCTGCTTGCTGATCCAC-3’.

### Cell proliferation assay

An MTT (Sigma–Aldrich) assay was performed according to the method described previously [21]. The PC-3 cells were seeded at a density of 5 × 10^3^ cells/well (DU145 cell, 3 × 10^3^ cells/well) into a 96-well culture plate and cultured in incubator for different lengths of time. Cell viability was measured by MTT assay as follows: 100 μl MTT-medium mixed solution (0.5 mg/ml) was added to each well and incubated with cells at 37°C for 4 h. The MTT solution was then discarded and 200 μl dimethyl sulfoxide (DMSO) was added to dissolve the formazan sediment. Finally the optical density was detected using a microplate reader (Molecular Devices) at an absorption wavelength of 490 nm. For colony formation assay, the cells were inoculated into 6-well culture plate with 500 cells per well and cultured for 7 d. Then the cells were stained with Crystal Violet and colonies containing.

### Cell migration assays

For transwell assay, the cells were seeded into transwell chambers (Corning Life Sciences) without FBS and chambers were set into 24-well plates with medium (with 10% FBS). Cells on small chambers were fixed by paraformaldehyde in 24 h or 48 h and stained with crystal violet, imaged, and counted. For wound healing assay, the cells (5 × 10 ^5^ cells/well) were inoculated into a 6-well plate and incubated for 24 h. A wound in each well was created by scraping a gap with a micropipette tip. After rinsing with PBS three times, cells were cultured with serum-free medium. The wound were recorded immediately and 12 h later (PC-3 cells, 24 h) under a microscope (Olympus) for DU145 cells.

### Antibodies and immunoblotting

The antibodies purchased were as follows: mouse anti-GAPDH antibody, rabbit anti-phospho-ERK1/2 and anti-phospho-AKT (Ser473) antibody from Cell Signaling Technology; anti-total ERK1/2 and anti-total AKT antibody from Santa Cruz Biotechnology; The protocol for immunoblotting has been described previously [13].

### Nude mice xenograft model

All animals were maintained and used in accordance with the guidelines of the Institutional Animal Care and Use Committee of the Institute for Nutritional Sciences. All of the experimental procedures were carried out in accordance with the Chinese Academy of Sciences ethics commission with an approval number 2010-AN-8. Mice were maintained on a 12-h light/dark cycle at 25°C. The four PC-3 cell lines were contributed, including PAQR3 knockdown cell, PAQR3 overexpression cell and control cell lines for both knockdown and overexpression respectively. The cells in the logarithmic phase of growth were trypsinized, centrifuged and rinsed with PBS three times. Each five nude mice (4 weeks old, male) were injected with a clonal population of PC-3 cell (5 × 10^6^ cells) in 0.1 ml of PBS in the upper right shoulders subcutaneous. Xenograft tumor sizes were measured by measuring two perpendicular diameters with digital calibers every other day and calculated according to the formula: 0.5 × length × width^2^.

### Statistical analysis

Statistical significance was assessed using *Student*'s *t* test. All results were expressed as the mean ± standard deviation (SD). Values of *P* < 0.05 were considered statistically significant.

## SUPPLEMENTARY MATERIALS FIGURES


