# Supplementary material for: PAQR3 suppresses the proliferation, migration and tumorigenicity of human prostate cancer cells
Source: Oncotarget. 2016 Jun 3;8(33):53948–58. doi: 10.18632/oncotarget.9807 (PMC5589553; doi:10.18632/oncotarget.9807)
Supplement: Supplementary file 1 [file oncotarget-08-53948-s001.pdf]

PAQR3 suppresses the proliferation, migration and tumorigenicity of human prostate cancer cells

Supplementary Materials

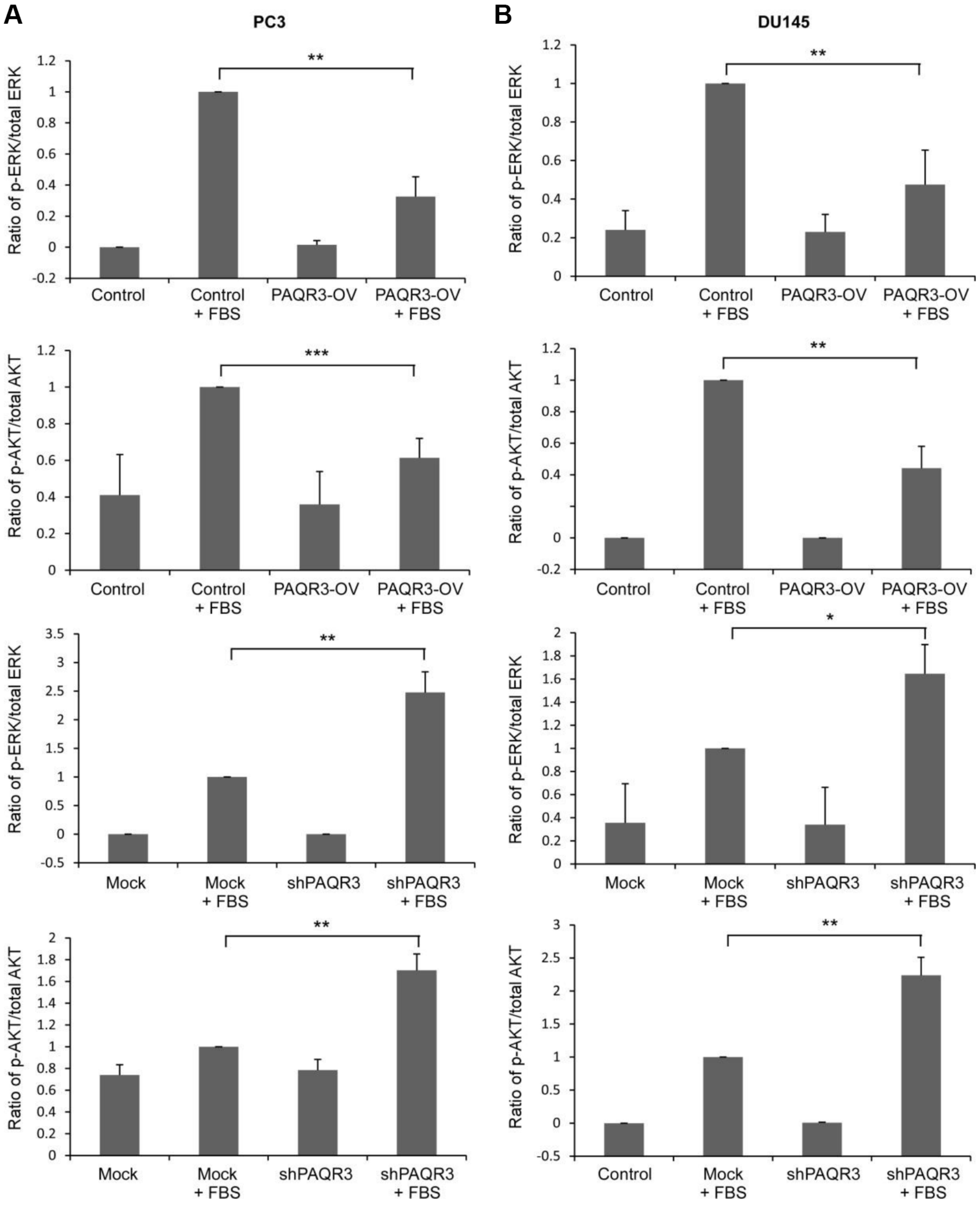

Supplementary Figure S1: Quantitative analysis of the Western blots in Figures 5A–5D. The Western blots of Figures 5A to 5D (three independent experiments for each dataset) were used to obtain quantitative values by Image-Pro Plus software. The data are shown as mean ± SD. * for $P < 0.05$, ** for $P < 0.01$ and *** for $P < 0.001$ between the groups as indicated.

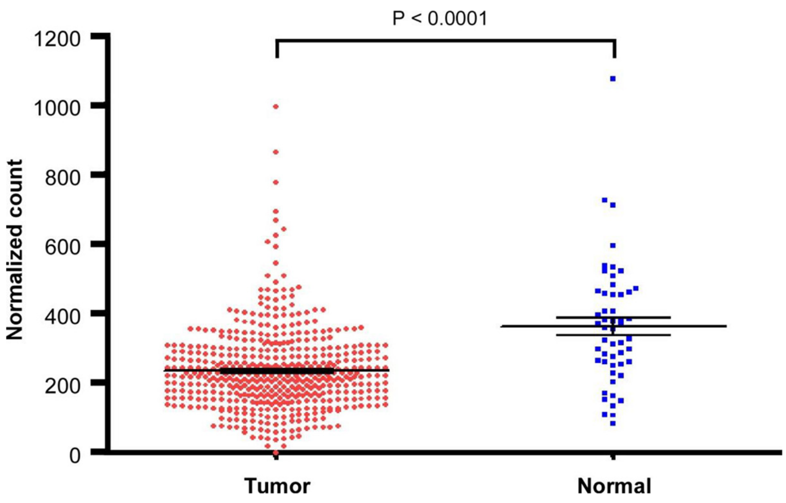

Supplementary Figure S2: The expression of PAQR3 is reduced in human prostate cancers. SeqV2 level 3 data for prostate adenocarcinoma samples were downloaded from TCGA Data Portal (https://tcga-data.nci.nih.gov). To date, there are 498 prostate adenocarcinoma samples and 52 normal prostate tissue samples in TCGA database. The mRNA expression data for PAQR3 was extracted and normalized count was used for comparison of their mRNA expression levels. The expression level of PAQR3 is significantly lower in prostate cancers than the normal tissues, indicating that PAQR3 may function as a tumor suppressor in the development of human prostate cancers.
